# Characterization of a variant of gap junction protein α8 identified in a family with hereditary cataract

**DOI:** 10.1371/journal.pone.0183438

**Published:** 2017-08-21

**Authors:** Debbie S. Kuo, Jared T. Sokol, Peter J. Minogue, Viviana M. Berthoud, Anne M. Slavotinek, Eric C. Beyer, Douglas B. Gould

**Affiliations:** 1 Department of Ophthalmology, University of California, San Francisco School of Medicine, San Francisco, CA, United States of America; 2 Department of Ophthalmology, Palo Alto Medical Foundation, Palo Alto, CA, United States of America; 3 Pritzker School of Medicine, University of Chicago, Chicago, IL, United States of America; 4 Department of Pediatrics, University of Chicago, Chicago, IL, United States of America; 5 Department of Pediatrics, University of California San Francisco, San Francisco, CA, United States of America; 6 Department of Anatomy and Institute of Human Genetics, University of California, San Francisco School of Medicine, San Francisco, CA, United States of America; University of Iowa, UNITED STATES

## Abstract

**Purpose:**

Congenital cataracts occur in isolation in about 70% of cases or are associated with other abnormalities such as anterior segment dysgenesis and microphthalmia. We identified a three-generation family in the University of California San Francisco glaucoma clinic comprising three individuals with congenital cataracts and aphakic glaucoma, one of whom also had microphthalmia. The purpose of this study was to identify a possible causative mutation in this family and to investigate its pathogenesis.

**Methods:**

We performed exome sequencing and identified a putative mutation in gap junction protein α8 (*GJA8*). We used PCR and DNA sequencing of *GJA8* in affected and unaffected members of the pedigree to test segregation of the variant with the phenotype. We tested cellular distribution and function of the variant protein by immunofluorescence and intercellular transfer of Neurobiotin in transiently transfected HeLa cells.

**Results:**

Exome sequencing revealed a variant in *GJA8* (c.658A>G) encoding connexin50 (Cx50) that resulted in a missense change (p.N220D) in transmembrane domain 4. The variant was present in all three affected family members, but was also present in the proband's grandfather who was reported to be unaffected. The mutant protein localized to the plasma membrane and supported intercellular Neurobiotin transfer in HeLa cells.

**Conclusions:**

We identified a variant in transmembrane domain 4 of Cx50 in a family with autosomal dominant congenital cataracts. This variant has been previously identified in other cataract cohorts, but it is also present in unaffected individuals. Our study demonstrates that the mutant protein localized to the plasma membrane and formed functional intercellular channels. These data suggest that *GJA8* c.658A>G is most likely a benign rare variant.

## Introduction

Cataracts form when crystalline lenses lose transparency and become opaque. Worldwide, cataracts are a major cause of treatable visual impairment in children, occurring in 1.2–6 per 10,000 live births [1]. Hereditary congenital cataracts account for 10–25% of pediatric cataract cases [2]. In these cases, cataracts can occur in isolation or with other ocular and systemic abnormalities. Mutations in lens crystallins and gap junction proteins constitute one of the most common genetic alterations causing hereditary congenital cataract, but there is considerable genetic heterogeneity [3].

Ocular lenses are avascular structures that derive nutrients from the adjacent aqueous humor. Water and solutes enter the lens driven by pumps in the epithelium, move through extracellular spaces, and cross the cell membranes of lens fiber cells before being brought back to the surface through channels contained in gap junctions; this process allows for nutrient and metabolite exchange, maintenance of resting potentials, and lens clarity [4,5]. Gap junction proteins (i.e., connexins) oligomerize to form hexameric hemichannels that traffic to the plasma membrane. Two hemichannels from adjacent cells dock with each other to form a gap junction channel containing a central pore that allows for communication between the cells. Connexins share a similar membrane topology with four transmembrane domains, two extracellular loops, one cytoplasmic loop, and cytoplasmic N- and C-termini [6]. In human lenses, three different gap junction proteins have been identified; mutants of those found in mature lens fiber cells, connexin50 (Cx50) and connexin46 (Cx46) have been associated with congenital cataracts [6].

In this study, we identified a family with congenital cataracts associated with a variant of *GJA8* encoding a substitution in the fourth transmembrane domain of Cx50 (c.658A>G; p.N220D) and examined the biochemical and functional consequences of this variant.

## Materials and methods

### Subjects

This study was approved by the institutional review board at the University of California, San Francisco (UCSF). Written informed consent was obtained from all participants or their parents (for children under 18 years of age). Medical records were reviewed. Subjects provided peripheral venous blood or saliva samples (Oragene Discover ORG-500, DNA Genotek, Ottawa, Ontario, Canada), and genomic DNA was extracted according to the manufacturer’s instructions (DNeasy Blood & Tissue Kit, Qiagen, Valencia, CA, USA).

### Genetic analysis

#### Exome sequencing

DNA samples were sent to the UCSF Institute for Human Genetics Genome core for library preparation, exome capture, and sequencing. Briefly, genomic DNA was sheared using a Covaris S2 sonicator (Woburn, Massachusetts, USA) to a target size of 200–300 base pairs, and assembled into a library with TruSeq adapters containing indexes that differentiate different libraries in a capture reaction as well as a sequencing run (KAPA Library Preparation Kit, Kapa Biosystems, Wilmington, MA, USA). Libraries were pooled into a capture reaction that contained biotinylated DNA oligonucleotides (‘baits’) from Nimblegen, (SeqCap EZ Human Exome Library v3.0; Roche Nimblegen, Madison, WI, USA) for 72 hours. The DNA bait-DNA hybrids were pulled out of the complex mixture by incubation with streptavidin-labeled magnetic beads and captured onto a strong magnet. After washing, the targeted DNA of interest was eluted and subjected to 18 cycles of DNA amplification. The sample was then sequenced using an Illumina HiSeq2000 sequencer (Illumina, San Diego, CA, USA). The data were filtered using GeneTalk (http://www.gene-talk.de) [7]. Variants were analyzed with Polyphen-2 (http://genetics.bwh.harvard.edu/pph2/) and SIFT (Sorting Intolerant From Tolerant Web Server, version 5.1.1, database release February 3, 2015; http://sift.bii.a-star.edu.sg/)to predict pathogenicity) [8,9].

#### Polymerase Chain Reaction (PCR) genotyping

DNA samples were genotyped using PCR. Taq Polymerase (Thermo Scientific, Waltham, MA, USA) was used to amplify the DNA according to the manufacturer’s directions using forward (5'-AGGCACTAAGAAGTTCCGGC -3') and reverse (5'- CAACCTCGGTCAAGGGGAAA-3') primers. The thermocycler was run at 94°C for 3 minutes, then cycled 40 times at 94°C for 30 seconds, 55°C for 30 seconds, and 72°C for 60 seconds, before final extension at 72°C for 5 minutes. The PCR product was purified with ExoSAP-IT (Affymetrix, Santa Clara, CA, USA). Then 10 ng of the PCR product was diluted in sterile water to 15 μl total volume with 8 pmol of primer (forward or reverse) for DNA sequencing (ELIM Biopharmaceuticals, Inc.; Hayward, CA, USA) in both directions.

### Molecular analysis

#### Generation of Cx50 constructs

The coding region of the wild-type human *GJA8* gene had been previously subcloned into pcDNA3.1/Hygro(+) (Life Technologies, Waltham, MA, USA) [10]. A corresponding plasmid encoding the *GJA8* mutant (c.658A>G; Cx50^N220D^) was obtained by PCR using High Fidelity Phusion DNA polymerase (New England BioLabs, Ipswich, MA, USA) [11]. Primers were designed in opposite directions to incorporate the 658A>G mutation into the PCR product (sense: 5'-GTGATGGAGTTGGGCCACCTGGGC-3' and antisense: 5'-GTCGAGGAATAGGGACACAGAGGCCAC-3'). The coding region of the construct was fully sequenced at the University of Chicago Comprehensive Cancer Center DNA Sequencing and Genotyping Facility to ensure that PCR amplification did not introduce unwanted mutations.

#### Cell culture and transfection

Communication-deficient HeLa cells were grown in MEM supplemented with non-essential amino acids, 10% fetal bovine serum, 2 mM glutamine, 100 units/ml penicillin G and 10 μg/ml streptomycin sulfate. Cells at 50% confluence were transiently transfected with wild type Cx50 in pcDNA3.1/Hygro(+) or with Cx50^N220D^ in pcDNA3.1/Hygro(+) using Lipofectin (Life Technologies, Carlsbad, CA, USA).

#### Immunoblotting and immunofluorescence

Immunoblotting to detect Cx50 and immunofluorescent localization of Cx50 in cultured cells were performed essentially as described previously [10,12]. Immunolocalization of Cx50 was assessed in many microscopic fields in three independent experiments. Photomicrographs were obtained using a Zeiss Axioplan 2 microscope (Carl Zeiss, Munich, Germany) equipped with a mercury lamp and a digital camera.

#### Intercellular transfer of gap junction tracers

Intercellular communication was assessed by microinjection of low molecular weight tracers as previously described [12]. Briefly, one cell within a cluster was microinjected for 1 min with a solution containing 5% Lucifer yellow (charge -2; MW: 444.4) and 9% Neurobiotin (charge: +1; MW: 287.2; Vector Laboratories) using a picospritzer (model PLI-188; NikonInstruments Inc., Melville, NY). After 5 min following microinjection of the tracers, cells were fixed in 4% paraformaldehyde, subjected to immunofluorescence (using rabbit anti-Cx50 antibodies and Alexa 488-conjugated goat anti-rabbit IgG antibodies), and incubated with Cy3-streptavidin conjugate to allow detection of the Neurobiotin by fluorescence microscopy. Lucifer yellow was used to identify the injected cell, because Cx50 shows limited permeability to this dye. Incidence of coupling was quantified in clusters of Cx50-immunopositive cells as the number of injections that resulted in Neurobiotin transfer divided by the number of injections.

## Results

**Fig 1:**
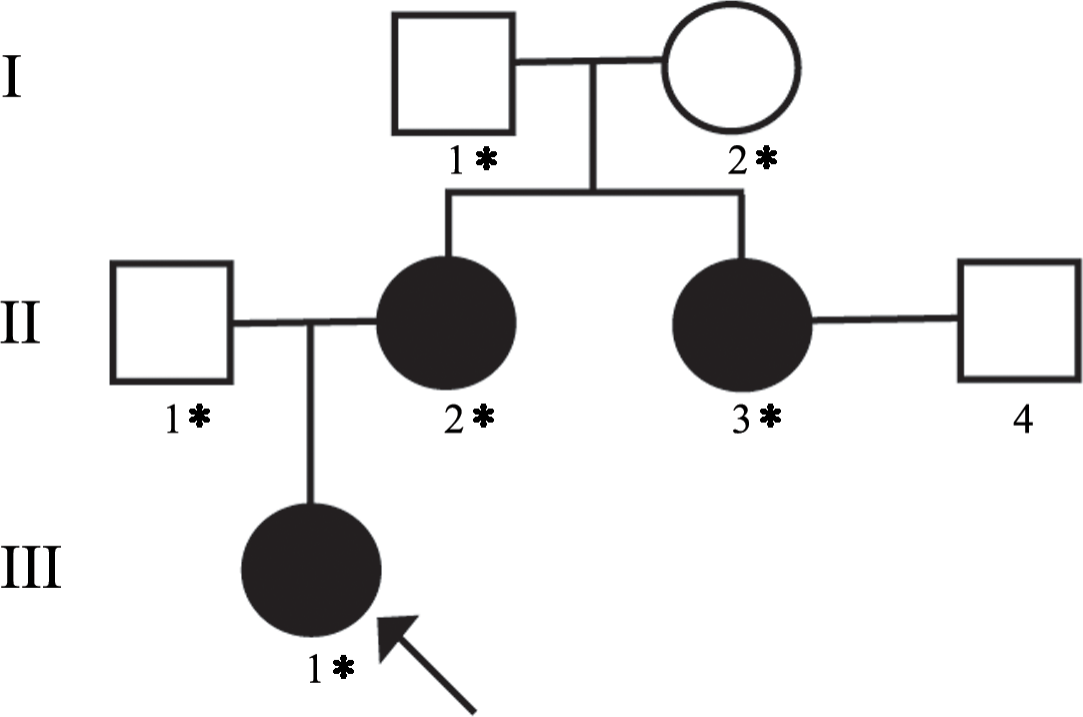
Pedigree of the family with autosomal dominant congenital cataract. The proband (III-1) is denoted with an arrow. Subject II-3 was also affected by microphthalmia. Asterisks indicate subjects from whom DNA was available.

We identified a three-generation Caucasian family comprising three individuals with congenital cataracts and aphakic glaucoma inherited in a pattern consistent with autosomal dominant transmission (**Fig 1**). The proband (III-1) was born 5 weeks prematurely and had a 14-day stay in the neonatal intensive care unit. She was noted to have partial cataracts at 4 weeks of age, which became very dense nuclear and cortical cataracts by 6 weeks of age. She underwent uncomplicated bilateral lensectomy and anterior vitrectomy at 7.5 weeks of age. She was diagnosed with aphakic glaucoma at 3 months of age and was treated with a glaucoma drainage tube in the right eye and aqueous suppressant medications in the left eye. The proband’s mother (II-2) was diagnosed with bilateral cataracts at a few months of age. She had bilateral cataract extraction at age 5 months and subsequently developed aphakic glaucoma in both eyes. The proband’s maternal aunt (II-3) had bilateral congenital cataracts and microphthalmia. She developed aphakic glaucoma in both eyes following cataract extraction, requiring multiple surgical interventions. The family’s medical history was otherwise unremarkable.

**Fig 2:**
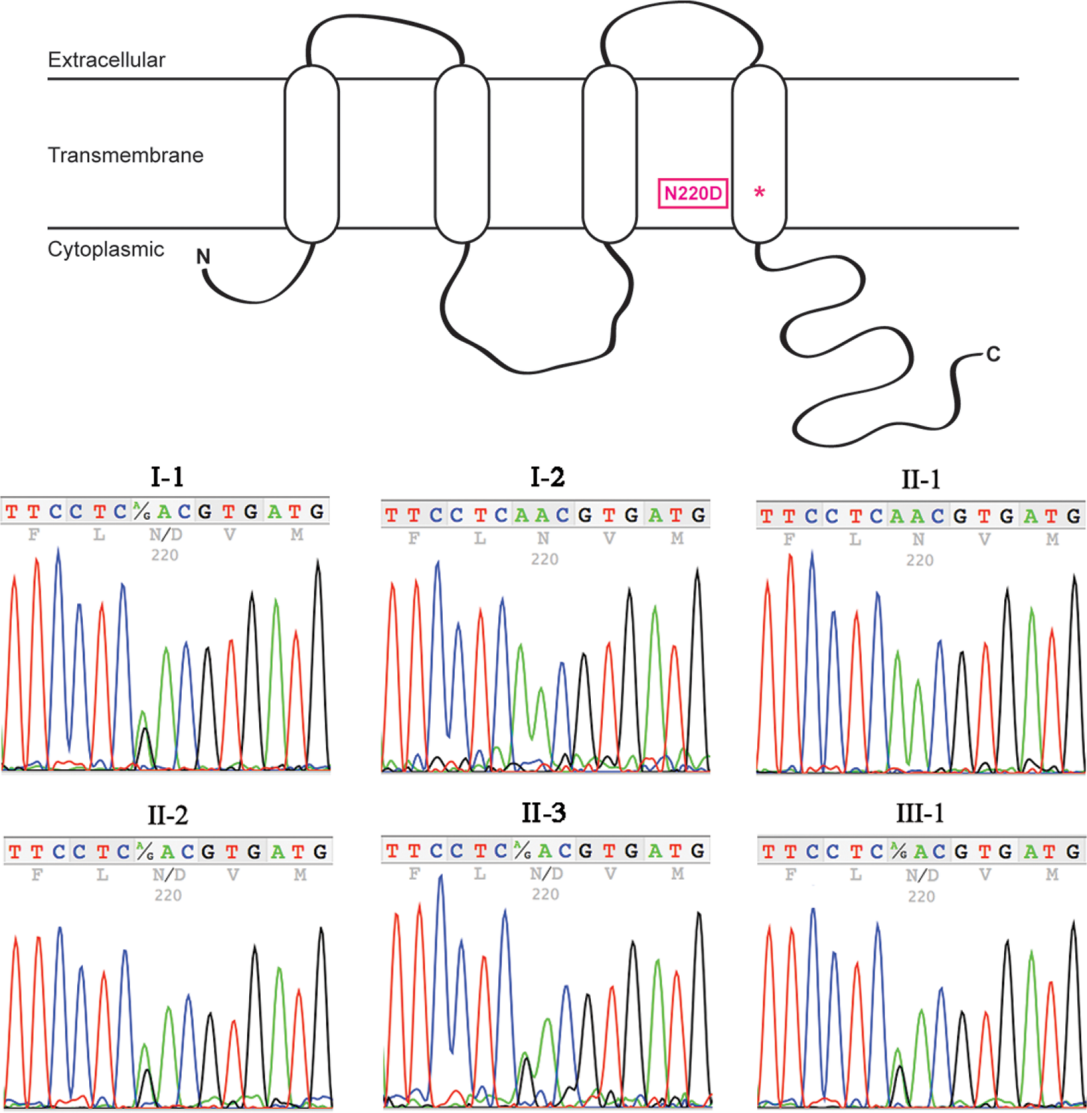
*GJA8* DNA sequencing. The c.658A>G variant results in missense mutation N220D within the fourth transmembrane domain of Cx50. PCR of *GJA8* and DNA sequencing revealed that the 658A>G variant was present in all affected subjects, III-1, II-2, and II-3. The variation was also present in the proband’s maternal grandfather (I-1).

**Fig 3:**
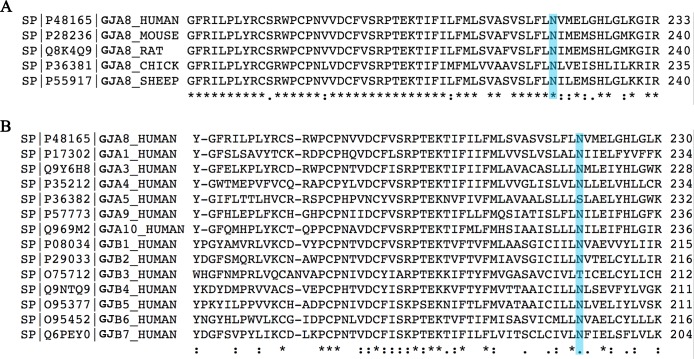
Protein sequence alignment. (A) Orthologous sequences of Cx50 from human, mouse, rat, chick and sheep show that amino acid N220 is highly conserved across species. (B) Alignment of other human gap junction protein sequences also demonstrated conservation of this residue among isoforms.

To investigate the causative mutation in this family, we first performed exome sequencing on DNA from subject II-2, which identified 66,151 variants. We filtered the results to allow only certain protein consequences (nonsynonymous, insertion/deletion, splice site affecting), leaving 12,118 variants. We then limited allele frequencies to <1% compared to public databases (1000 Genomes, Exome Sequencing Project, Exome Aggregation Consortium), resulting in 1,223 variants. We searched for mutations within candidate genes (**Table 1**) that have been associated with autosomal dominant cataract in multiple studies. Only one of the remaining variants was present within a candidate gene: *GJA8*c.658A>G, which leads to a missense mutation p.N220D within the fourth transmembrane domain. When we tested for segregation of this variant with the phenotype we found that it was present in each of the individuals with cataracts, but it was also found in the proband's maternal grandfather (subject I-1) (**Fig 2**). This individual was reported by the family to have had no congenital cataracts; however, he was not available for examination. The *GJA8* c.658A>G variant has a population frequency of 0.005 (1000 Genomes, Exome Sequencing Project). The asparagine residue at position 220 is highly conserved across species and isoforms (**Fig 3**) and is predicted to be pathogenic by SIFT (score 0.01 out of 1.00, “damaging”) and Polyphen-2 (score 0.999 out of 1.000, “probably damaging”).

**Table 1:** List of candidate genes for hereditary cataract.

**Gene**	**Name**	**Exons (#)**
AQP0/MIP	aquaporin/major intrinsic protein of lens fiber	4
BFSP2	beaded filament structural protein 2	7
CHMP4B	charged multivesicular body protein 4b	5
CRYAA	crystallin, alpha A	3
CRYAB	crystallin, alpha B	3
CRYBA1	crystallin, beta A1	6
CRYBA2	crystallin, beta A2	4
CRYBA3	crystallin, beta A3	6
CRYBA4	crystallin, beta A4	6
CRYBB1	crystallin, beta B1	6
CRYBB2	crystallin, beta B2	6
CRYBB3	crystallin, beta B3	6
CRYGB	crystallin, gamma B	3
CRYGC	crystallin, gamma C	3
CRYGD	crystallin, gamma D	3
CRYGS	crystallin, gamma S	3
Cx46/GJA3	connexin 46/gap junction alpha-3	2
Cx50/GJA8	connexin 50/gap junction alpha-8	2
EYA1	eyes absent homolog 1 (Drosophila)	18
FOXE3	forkhead box E3	1
FTL	ferritin, light polypeptide	4
HSF4	heat shock transcription factor 4	15
MAF	v-maf avian musculoaponeurotic fibrosarcoma oncogene homolog	1
PAX6	paired box gene 6	15
PITX3	pituitary homeobox 3	4
WFS1	Wolframin	8

**Fig 4:**
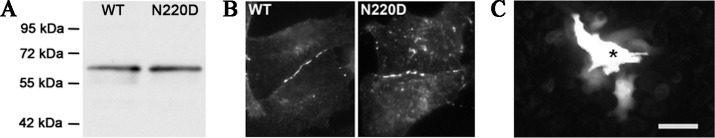
Cx50^N220D^ production, localization and function in transfected cells. (A) Proteins from homogenates of HeLa cells transiently transfected with wild type Cx50 or Cx50^N220D^ were resolved by SDS-PAGE and subjected to immunoblotting using rabbit polyclonal anti-human Cx50 antibodies. Samples from cells expressing either Cx50 or Cx50^N220D^ contain a single immunoreactive band of similar mobility. Molecular mass standards are indicated on the left. (B) Photomicrographs show the distribution of immunoreactivity to anti-Cx50 antibodies in HeLa cells transfected with wild type Cx50 (WT) or Cx50^N220D^ (N220D). Cells expressing WT and N220D both show substantial labeling at appositional interfaces between cells consistent with gap junction plaques. (C) Photomicrograph showing intercellular neurobiotin transfer from a microinjected cell (*) to neighboring cells in HeLa cells transiently with CX50^N220D^. Bar, 16 μm for B and 10 μm for C.

We then examined the consequences of this variant using communication-deficient HeLa cells transiently transfected with wild type *GJA8* (encoding wild type Cx50) or with the c.658A>G allele (encoding Cx50^N220D^). Immunoblots of homogenates from HeLa cells transiently transfected with wild type Cx50 or Cx50^N220D^ showed an immunoreactive Cx50 band of similar electrophoretic mobility (**Fig 4A**). To test the ability of the mutant protein to form gap junction plaques, we examined the cellular distribution of the proteins by immunofluorescence. Cells transfected with wild type Cx50 and Cx50^N220D^ had substantial labeling at appositional membranes (**Fig 4B**) indicating successful trafficking. To test the ability of Cx50^N220D^ to form functional intercellular channels, we microinjected cells after transfection with the gap junction permeant tracer, Neurobiotin. Both wild type Cx50 and the Cx50^N220D^ variant transferred Neurobiotin to their neighboring cells (**Fig 4C**); the incidence of coupling in wild type Cx50-transfected cells was 9/9 and in Cx50^N220D^-transfected cells was 10/11.

## Discussion

We identified a variant of *GJA8* (c.658A>D) in a family with autosomal dominant cataract. This variant results in the substitution of a polar, uncharged asparagine at amino acid position 220 for a negatively charged aspartic acid (N220D). Asparagine at this position in Cx50 is highly conserved across species and across other connexin isoforms. The substitution is predicted to be probably damaging based on bioinformatic algorithms. However, based upon genetic evidence, the pathogenicity of this variant is equivocal. It was first identified in a subject from a family with congenital cataracts, but was also found in 1 out of 340 alleles in the control population (allelic frequency: 0.003), which led to the conclusion that was a non-pathogenic polymorphism [13]. The mutation was also found in a subject with congenital cataract and microcornea and in his unaffected father, but not in his affected sibling, indicating that it did not segregate with the disease in the family [14]. In our pedigree, the mutation was present in all affected subjects, but it was also found in the proband’s maternal grandfather who was reported to be unaffected. These observations suggest that the mutation is either an incidental finding or that it is pathogenic with reduced penetrance and variable expressivity. Environmental factors, stochastic events, epigenetic changes or differences in genetic background (or modifiers) can all contribute to differential effects of a mutation. Notably, in the family reported here all affected members are female, however, in a previous study the affected child and the unaffected father that carried the mutation were male [14].

Ours is the first study to characterize the consequences of the N220D variant on the biochemical and functional properties of Cx50. The most common pathogenic mechanism found in other cataract-associated Cx50 mutants is failure to form gap junctions due to impaired protein folding and/or localization to the plasma membrane [6,15,16]. Unlike most pathogenic mutations, Cx50^N220D^ localized to appositional plasma membranes and formed functional gap junction plaques between adjacent cells. These results demonstrate that this variant does not abolish intercellular channel function (at least not in transfected HeLa cells). Variations of cell density within the same tissue culture dish and of protein expression in transient transfection experiments preclude us from estimating whether the extent of transfer differs between Cx50 and Cx50^N220D^. The ability of Cx50^N220D^ to support intercellular communication may explain the lack of segregation of the variant with the cataract phenotype in the different families. The results suggest that other factors might contribute to the pathology.

Although Cx50^N220D^ is the only variant in the fourth transmembrane domain of Cx50 identified to date, mutations in the corresponding region of other connexins have been associated with diseases, including autosomal dominant congenital cataract (Cx46^F206I^) and autosomal dominant and recessive hearing loss (Cx26^C202F^ and Cx26^N206S^, respectively). Interestingly, the N220D substitution in Cx50 occurs in a homologous position to N206S in Cx26, however, the functional consequences appear to be different. This might relate to the differential permeability of the intercellular channels formed by Cx50 and Cx26. Cx50 channels are permeable to Neurobiotin, but show limited intercellular transfer of Lucifer yellow whereas Cx26 channels are permeable to both cationic and anionic molecules with a cationic preference. This implies that the amino acid residues determining permeation must differ between Cx26 and Cx50 [17–21]. N206 is involved in critical interactions within the Cx26 channel; the crystal structure of Cx26 shows that N206 forms a hydrogen bond with R143, located on the third transmembrane domain [22]. Mutations in this residue are predicted to disrupt this interaction; indeed, Cx26^N206S^ gap junction channels show altered voltage gating properties and decreased permeability to ethidium (charge +1, MW: 314.39), but preserve permeability to anionic Lucifer yellow [18,20]. In Cx50 ^N220D^, however, we did not find a difference in intracellular transfer of Neurobiotin compared to wild-type Cx50.

In summary, we identified and characterized a variant occuring in transmembrane domain 4 of Cx50 in a family with autosomal dominant congenital cataracts. Functional studies demonstrated that Cx50^N220D^ forms gap junctions that allow the passage of small, cationic molecules, similar to wild-type Cx50, supporting that this is a rare, benign variant rather than the causative mutation in this family.
